# Selection of competent blastocysts for transfer by combining time-lapse monitoring and array CGH testing for patients undergoing preimplantation genetic screening: a prospective study with sibling oocytes

**DOI:** 10.1186/1755-8794-7-38

**Published:** 2014-06-22

**Authors:** Zhihong Yang, John Zhang, Shala A Salem, Xiaohong Liu, Yanping Kuang, Rifaat D Salem, Jiaen Liu

**Affiliations:** 1 ART and REI Division, Pacific Reproductive Center, Torrance, CA, USA; 2 ART Division, New Hope Fertility Center, New York, NY, USA; 3 IVF and REI Division, Jia En De Yun Hospital, Beijing, People’s Republic of China; 4 ART Department, Ninth People’s Hospital, Shanghai Jiao Tong University School of Medicine, Shanghai, People’s Republic of China; 5 ZytoGen Global Genetics Institute, Timonium, MD 21093, USA

**Keywords:** Time-lapse monitoring, Array CGH, PGS, Ploidy, Implantation, Miscarriage

## Abstract

**Background:**

Recent advances in time-lapse monitoring in IVF treatment have provided new morphokinetic markers for embryonic competence. However, there is still very limited information about the relationship between morphokinetic parameters, chromosomal compositions and implantation potential. Accordingly, this study aimed at investigating the effects of selecting competent blastocysts for transfer by combining time-lapse monitoring and array CGH testing on pregnancy and implantation outcomes for patients undergoing preimplantation genetic screening (PGS).

**Methods:**

A total of 1163 metaphase II (MII) oocytes were retrieved from 138 PGS patients at a mean age of 36.6 ± 2.4 years. These sibling MII oocytes were then randomized into two groups after ICSI: 1) Group A, oocytes (n = 582) were cultured in the time-lapse system and 2) Group B, oocytes (n = 581) were cultured in the conventional incubator. For both groups, whole genomic amplification and array CGH testing were performed after trophectoderm biopsy on day 5. One to two euploid blastocysts within the most predictive morphokinetic parameters (Group A) or with the best morphological grade available (Group B) were selected for transfer to individual patients on day 6. Ongoing pregnancy and implantation rates were compared between the two groups.

**Results:**

There were significant differences in clinical pregnancy rates between Group A and Group B (71.1% vs. 45.9%, respectively, *p* = 0.037). The observed implantation rate per embryo transfer significantly increased in Group A compared to Group B (66.2% vs. 42.4%, respectively, *p* = 0.011). Moreover, a significant increase in ongoing pregnancy rates was also observed in Group A compared to Group B (68.9% vs. 40.5%. respectively, *p* = 0.019). However, there was no significant difference in miscarriage rate between the time-lapse system and the conventional incubator (3.1% vs. 11.8%, respectively, *p* = 0.273).

**Conclusions:**

This is the first prospective investigation using sibling oocytes to evaluate the efficiency of selecting competent blastocysts for transfer by combining time-lapse monitoring and array CGH testing for PGS patients. Our data clearly demonstrate that the combination of these two advanced technologies to select competent blastocysts for transfer results in improved implantation and ongoing pregnancy rates for PGS patients.

## Background

Since the inception of in vitro fertilization, selection of the most competent embryos for transfer has been a primary focus of investigation. As the field progressed, an increasing number of studies have concentrated on developing more advanced technologies, both invasive and non-invasive, to select the most competent embryos with the highest potential of implantation for transfer. The original non-invasive technique, morphological grading has long been a primary method of evaluating and selecting embryos for transfer [1-3]. Traditional procedures for embryo evaluation and selection are based on the morphological characteristics observed with a microscope at several discrete time points of embryonic development. At the early cleavage stage, morphological parameters including cell number, proportion of fragmentation, presence and number of nuclei, size and symmetry of blastomeres are used to evaluate and select embryos for transfer [1-9]. At the blastocyst stage, the degree of blastocyst expansion and morphology of inner cell mass (ICM) and trophectoderm (TE) are commonly used to evaluate and grade the blastocysts [2,10]. Nevertheless, transfer of the top grade embryos often fails to establish a viable pregnancy, while replacement of embryos with poor morphological scores sometimes results in a live birth. Thus, there are obvious shortcomings with traditional methods of evaluation and selection of embryos for transfer based on morphological characteristics alone [11-14]. In addition, morphological evaluation usually requires observation of embryos outside of a conventional incubator. When embryos are evaluated under a microscope in an uncontrolled environment, they may suffer from undesirable shock or stress due to sudden changes in known critical parameters including temperature, oxygen concentration and pH [15-19].

The adverse effects associated with using conventional incubators have limited the frequency of microscopic evaluation of embryos, as only limited information about growth and changes in embryonic morphology can be obtained at a few discrete time points. The recent development of time-lapse culture and monitoring has overcome this limitation by combining incubation and observation of embryos into one unique system [20-25]. As a result, time-lapse monitoring has gradually emerged as one of the most advanced non-invasive methods for evaluation of embryonic competence [20-36]. A retrospective cohort study with logistic regression analysis of a large sample size (n = 7305) concluded that embryo incubation and selection in the time-lapse system significantly improved pregnancy and implantation rates compared to the conventional incubator [34]. In contrast, other studies have concluded that there were no significant differences in clinical pregnancy outcomes between the time-lapse system and the conventional incubator [32,33]. However, the chromosomal compositions of the transferred embryos had not been determined when they were selected for transfer to patients in any of these studies.

It has been well documented that the main cause of embryo arrest, implantation failure and pregnancy loss is the presence of numerical chromosome abnormality or aneuploidy [11,37-43]. Aneuploidy is the most common abnormality in in vitro fertilized embryos [44-46], and increases with maternal age [39-41]. As the original invasive method for embryo selection, preimplantation genetic diagnosis (PGD) was initially applied to the screening of X-linked disorders [47] and monogenetic diseases with PCR [48,49], and later extended to aneuploidy screening with the use of fluorescence in situ hybridization (FISH) [50-53]. In early PGS studies, only a limited number of chromosomes (5–12) were screened using FISH, which had an error rate of 5-15% and resulted in disappointing pregnancy outcomes [54-57]. Conventional CGH was then introduced to screen all 24 chromosomes of oocytes and embryos with some success [58-62]. Array CGH [63-71], single nucleotide polymorphism (SNP) array [72-76] and PCR-based comprehensive chromosomal screening (CCS) [77-80] have been recently applied to the screening of embryos before transfer to improve the efficiency of IVF and PGD treatments. In these studies, polar body, blastomere and/or trophectoderm biopsies were performed and copies of all 24 chromosomes were analyzed within 24–48 hours. However, the biopsied oocytes and embryos in these studies were entirely cultured in the conventional incubator and the impact of culture conditions on embryo morphokinetics and implantation potential remains largely unexplored, although some delay in blastocyst development in vitro was observed following blastomere biopsy on day 3 [31]. More recently, an interesting model for classifying the risk of aneuploidy has been proposed based on morphokinetics of human embryos that were cultured in the time-lapse system [20]. However, pregnancy and implantation data from the study group of patients were unavailable in this report, although a retrospective study was performed to apply this risk model to analyze the outcomes of a group of IVF patients (without PGS) whose embryos were monitored in the time-lapse system [21]. To date, there is still very limited information regarding the efficiency of combining time-lapse monitoring and array CGH testing to select competent blastocysts for transfer in terms of pregnancy and implantation outcomes.

At present, there is no consensus on the best way to determine the competency of human embryos derived from in vitro fertilization or to select the most competent embryos for transfer despite the recent advances in both invasive and non-invasive techniques. In response to this challenge, our current study explores the use of both time-lapse monitoring and array CGH testing to select competent blastocysts for transfer in order to improve pregnancy and implantation rates for PGS patients. In particular, our approach for this study was to compare the effects of the time-lapse system and the conventional incubator on embryo ploidy and implantation potential in PGS patients using a sibling oocyte model.

## Methods

### Ethics statement

We obtained ethics approval for our study from the ethics committees (also known as an Institutional Review Board, IRB) at our respective institutions. All the participants had the capacity to consent and we obtained written informed consents from all patients enrolled in the current study.

### Inclusion and exclusion criteria for patients

Patients undergoing preimplantation genetic screening at our IVF clinics were offered enrollment in this IRB approved study from February to December of 2012. A written informed consent was obtained from all patients and pre-treatment counseling was provided to each couple. Standard clinical protocols and laboratory procedures were used for the treatment of all patients in this study. The cohort patients (n = 138) requested PGS with array CGH screening due to the following clinical indications: 1) unexplained recurrent pregnancy loss (URPL): patients (n = 71) with two or more unexplained miscarriages; 2) repeated implantation failure (RIF): patients (n = 32) with implantation failure after three or more IVF cycles or with transfer of 10 or more good morphology embryos; and 3) previous aneuploid conceptions (PAC): patients (n = 35) with one or more previous aneuploid conceptions (e.g. Down Syndrome). Patients were eligible for this study if they met the following inclusion criteria: 1) female patient’s age ≤ 39 years with normal karyotypes; 2) ≥ 8 oocytes retrieved; 3) presence of both ovaries and normal uterine lining; 4) undergoing preimplantation genetic screening for their embryos; and 5) willingness to participate in the study and to follow instructions. PGS patients whose treatment incorporated donor gametes or frozen and thawed embryos were not included. Patients with severe endometriosis or endometrial factors related infertility were excluded. Known translocation carriers (either parent) were also excluded from this study.

### Ovarian stimulation, oocyte retrieval and fertilization

All enrolled patients had an ultrasound scan and serum evaluation of FSH, LH and E_2_ on day 2 of their menses and were stimulated with conventional down-regulation protocols. In brief, patients were down regulated with Lupron and started stimulation on day 3 with r-FSH (Gonal-F, Sereno). The patients were monitored with serial transvaginal ultrasound and E_2_ levels to monitor their follicular growth and endometrial lining. When at least three follicles reached ≥19 mm in diameter, a single dosage of 250 μg recombinant hCG (Ovidrel, Sereno) was administered. For all patients, oocyte retrieval was performed under transvaginal ultrasound guidance at 35 to 36 hours after administration of hCG. After stripping of cumulus cells, oocytes at MII stage were inseminated with ICSI 4 hours after retrieval as previously described [12,49]. The microinjected oocytes from each patient were washed and pooled together in a culture dish containing 1 mL modified human tubal fluid (mHTF, Irvine Scientific, Irvine, USA) + 10% synthetic serum substitute (SSS, Irvine Scientific, Irvine, USA). The microinjected sibling oocytes were then randomized into two groups: 1) Group A: the microinjected oocytes were cultured in the time-lapse system (EmbryoScope™, Unisense FertiliTech, Aarhus, Denmark) at 37°C, 6% CO_2_, 5% O_2;_ and 2) Group B: the microinjected oocytes were cultured in the conventional incubator (Heraeus, Heracell*, Thermo Scientific, Waltham, MA, USA) at 37°C, 6% CO_2_, 20% O_2_. The conventional incubator was used only for culture of the randomized embryos throughout the entire study.

### Embryo culture, trophectoderm biopsy and array CGH analysis

For both groups, fertilization was assessed 16–18 hours post insemination by ICSI. All zygotes with two pronuclei and two polar bodies were considered normally fertilized. All embryos in the two groups were cultured from one-cell to blastocyst stage in a continuous single culture medium (CSC, Irvine Scientific, Irvine, USA) plus 10% SSS. For comparison, embryos in the two groups were cultured in the same type of culture dish (Embryoslide, Unisense FertiliTech, Denmark). Each well of the culture dish was filled with 20 μl of the culture medium and the slide was covered with 1.3 mL of light mineral oil (Irvine Scientific, Irvine, USA). All culture dishes were prepared and equilibrated at least 6 h prior to use. When embryos developed to the blastocyst stage on day 5, an opening of 6 to 9 um was made in the zona pellucida with two to three pulses of 19 ms from a non-contact 1.48 um diode Octax laser system (MTG, Bruckberg, Germany), and an average of 4 (3 to 5) trophectoderm (TE) cells were aspirated into a biopsy pipette and separated from the blastocysts by applying several laser pulses of 14 ms between the trophectoderm cells at the stretching area. The biopsied TE cells were washed in 1x PBS and loaded into a PCR tube containing 2.5 μl 1x PBS. All the biopsy and manipulation procedures were performed in a fully enclosed isolator-based workstation with temperature and gas control to provide a controlled environment for manipulation of embryos (Origio Mid Atlantic Devices, Mt. Laurel, USA).

Whole genomic amplification and array CGH testing were performed as previously described [12-14]. In brief, whole genomic amplification was performed using the SurePlex kit (BlueGnome, Cambridge, UK). The amplified sample DNA and control (normal male and female) DNA were labeled with Cy3 and Cy5 fluorophores for 2–4 hours. Labeled DNA was then re-suspended in a dexsulphate hybridization buffer and hybridized onto the 24 sure chips under cover slides for 4–6 hours. After washing and drying, the hybridized 24 sure chips were scanned at 10 μm using a laser scanner (Agilent, Sainte Rosa, USA). The data was analyzed using the BlueFuse Multi software (BlueGnome, Cambridge, UK) for whole chromosome gain or loss.

### Embryo evaluation and selection for transfer

In the conventional incubator group, fertilization was assessed at 16 to 18 hours post ICSI under a stereoscope, and the fertilized zygotes were then cultured to blastocyst stage as described above. Blastocysts were evaluated microscopically and graded according to the morphological criteria described elsewhere [10]. Blastocysts were graded from 1 to 6 based on their degree of expansion and hatching status: 1) Grade 1 or early blastocyst: the blastocoele is less than half of the volume of the embryo; 2) Grade 2 or blastocyst: the blastocoele is more than half of the volume of the embryo; 3) Grade 3 or full blastocyst: the blastocoele occupies the embryos completely; 4) Grade 4 or expanded blastocyst: the blastocoele is larger than the early embryo and the zona pellucida turns thinner; 5) Grade 5 or hatching blastocyst: trophectoderm cells start herniating from the zona pellucida; and 6) Grade 6 or hatched blastocyst: the blastocyst has escaped the zona pellucida completely. For blastocysts of Grades 3 to 6, inner cell mass (ICM) and trophetoderm (TE) were also evaluated and graded accordingly. The ICM was graded into three categories: A (many ICM cells packed together tightly), B (several ICM cells grouped loosely) and C (very few ICM cells). The trophectoderm was also graded into three categories: A (many trophectoderm cells forming a multiple epithelium layer), B (few trophectoderm cells consisting of a loose epithelium layer) and C (very few trophectoderm cells).

In the time-lapse system group, images of individual embryos were captured with a built-in digital camera every 20 minutes at 7 different focal planes. Fertilization was assessed at 16 to 18 hours post ICSI insemination according to the digital images acquired with the time-lapse monitoring system. Detailed analysis of the acquired images of each embryo was made with the EmbryoView software (Unisense FertliTech, Denmark), and all the targeted events related to embryonic development were then annotated together with the corresponding hour post ICSI insemination (hpi). All morphokinetic data were recorded as mean ± SD hpi.

In the time-lapse system group, embryo selection for transfer was primarily based on array CGH analysis. When multiple euploid blastocysts were recognized from individual patients, the morphokinetic markers were the secondary criterion for selection according to the most predictive parameters that are highly correlated with implantation as described elsewhere [26,34]. The most predictive parameters include (i) t5 = time of division to 5 cells: 48.8 - 56.6 hpi; (ii) cc2 = time between division to 2 cells and division to 3 cells (≤11.9 h) and (iii) s2 = time between division to 3 cells and subsequent division to 4 cells (≤0.76 h). One to two euploid blastocysts within the most predictive parameters were selected for transfer to individual patients according to the patient’s age and clinical indications on day 6. In the conventional incubator group, embryo selection was primarily based on the array CGH results. The morphological grading by microscopic evaluation was the secondary criterion for selection when multiple euploid blastocysts were available. One to two euploid blastocysts with the best morphological grade available were selected for transfer to individual patients on day 6. The surplus euploid blastocysts after embryo transfer in both groups were frozen for future FET cycles as previously described [12-14].

### Sample size calculation and statistical analysis

Sample size was calculated using GraphPad StatMate (GraphPad Software, San Diego, California, USA). Based on our previous clinical studies in which nearly 50% of all inseminated MII oocytes developed into blastocysts [12-14], a sample size of 500 MII oocytes had an 80% power to detect a difference between means of 0.20 with a significance level of 0.05 (two-tailed value). Clinical pregnancy, implantation and ongoing pregnancy rates were tabulated and compared between the time-lapse system and the conventional incubator groups. Clinical pregnancy was defined as an intrauterine gestational sac with fetal heartbeat visualized by ultrasound examination at week 8 after embryo transfer. Ongoing pregnancy was defined as continuing pregnancy at ≥ 20 weeks of gestation. Implantation rate was calculated as the total number of sacs with fetal heart beat over total embryos transferred. The categorical variables were analyzed by Chi-square analysis or Fisher’s exact test as appropriate. The time-lapse variables were first tested for normality using the Shapiro-Wilk test first and then analyzed by the Mann–Whitney test since the majority of the variables were normally distributed. The statistical analyses were performed using GraphPad InStat version 3.10 (GraphPad Software, San Diego, California, USA). A two-tailed value of *p* <0.05 was considered statistically significant.

## Results

**Figure 1:**
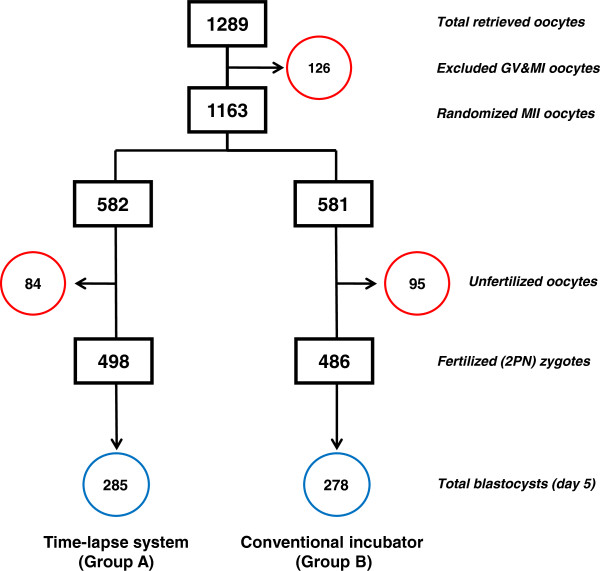
**Schematic for oocytes randomized into either the time-lapse system (Group A) or the conventional incubator (Group B).** GV = germinal vesicle; MI = metaphase I; MII = metaphase II; 2PN = two pronuclei; Excluded immature (GV and MI) oocytes and unfertilized oocytes in each group were circled in red. The total number of blastocysts associated with each group is circled in blue.

During the 10 month study period, a total of 138 (81.7%) patients who met all the inclusion criteria completed this study while 31 (18.3%) patients who had less than 8 oocytes were excluded from this study. A total of 1163 metaphase II (MII) oocytes were retrieved from 138 PGS patients at a mean age of 36.6 ± 2.4 years. 126 (9.8%) of the retrieved oocytes at germinal vesicle (GV) and/or metaphase I (MI) stages were excluded before randomization. 1163 (90.2%) oocytes at metaphase II (MII) stage were randomized into two groups after ICSI: 1) Group A, the microinjected oocytes (n = 582) were cultured in the time-lapse system, and 2) Group B, the microinjected oocytes (n = 581) were cultured in the conventional incubator (Figure 1). There was no significant difference in fertilization rate between Group A and Group B (85.6% vs. 83.6%, respectively, *p* >0.05). The blastocyst formation rate (per microinjected MII oocytes) on day 5 in Group A was also similar to that in Group B (48.9% vs. 47.8%, respectively, *p >*0.05) (Table 1).

**Table 1:** Comparison of fertilization and blastocyst formation rates between time-lapse system (Group A) and conventional incubator (Group B)

**Parameters**	**Group A**	**Group B**	***p *value**
Total MII oocytes	582	581	
Oocytes fertilized (2PN)	498 (85.6%)	486 (83.6%)	0.409^a^
Blastocysts	285 (48.9%)	278 (47.8%)	0.746^a^

MII = metaphase II; 2PN = two pronuclei; ^a^by Chi-square analysis.

**Figure 2:**
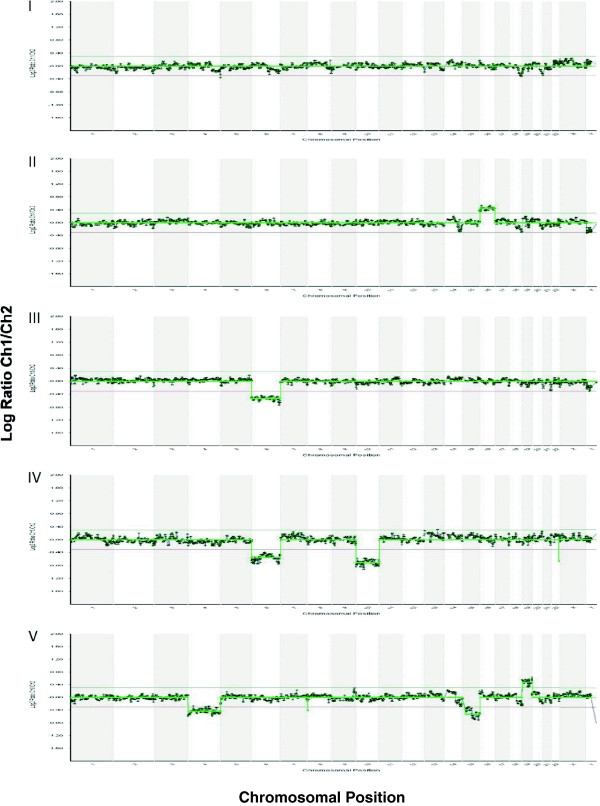
**Representative array CGH profiles showing euploidy and different types of aneuploidy detected in both Group A and Group B.** I. Euploid profile with normal chromosomal copies; II. Aneuploid profile with single chromosomal gain (trisomy): a gain of chromosome 16; III. Aneuploid profile with single chromosomal loss (monosomy): a loss of chromosome 6; IV. Aneuploid profile with dual chromosomal abnormalities: losses of chromosomes 6 and 10; V. Aneuploid profile with complex chromosomal abnormalities: a gain of chromosome 19 and losses of chromosomes 4 and 15.

In the time-lapse system group, 263 (92.3%) blastocysts were biopsied and analyzed by array CGH. Biopsies could not be completed for 22 (7.7%) blastocysts due to either poor morphology or degeneration after biopsy. Array CGH analysis revealed euploidy in 121 (46.0%) and aneuploidy in 135 (51.3%) of the biopsied blastocysts. No results occurred in 7 (2.7%) of the biopsied blastocysts due to DNA amplification failure. In the conventional incubator group, 265 (95.3%) blastocysts were biopsied while 13 (4.7%) blastocysts were not biopsied due to either poor morphology or degeneration after biopsy. Array CGH analysis revealed euploidy in 105 (39.6%), aneuploidy in 156 (58.9%) and no results in 4 (1.5%) of the biopsied blastocysts (Table 2). There was a non-significant trend towards more euploid embryos developing to the blastocyst stage in the time-lapse system group compared to the conventional incubator group (46.0% vs. 39.6%, respectively, *p >*0.05). Chromosomal abnormalities were detected in all 24 chromosomes in both Group A and Group B. All types of aneuploidies were observed in both Group A and Group B, including single chromosome gain (or trisomy), single chromosome loss (or monosomy), dual (two) and complex (three or more) chromosomal abnormalities (Figure 2). There were no significant differences in the proportions of each type of aneuploidy between the two groups (*p* >0.05) (Table 3).

**Table 2:** Comparison of biopsy and array CGH results between time-lapse system (Group A) and conventional incubator (Group B)

**Parameters**	**Group A**	**Group B**	***p *value**
Total blastocysts	285	278	
Biopsied blastocysts	263 (92.3%)	265 (95.3%)	0.135^a^
No results	7 (2.7%)	4 (1.5%)	0.545^b^
Euploid	121 (46.0%)	105 (39.6%)	0.163^a^
Aneuploid	135 (51.3%)	156 (58.9%)	0.098^a^

^a^by Chi-square analysis, ^b^by Fisher’s exact test.

**Table 3:** Comparison of array CGH results of aneuploid blastocysts between time-lapse system (Group A) and conventional incubator (Group B)

**Parameters**	**Group A**	**Group B**	***p *value**
Total aneuploid blastocysts	135	156	
Monosomy	31 (22.9%)	34 (21.8%)	0.922^a^
Trisomy	22 (16.3%)	23 (14.7%)	0.839^a^
Dual chromosomal abnormality	36 (26.7%)	41 (26.3%)	0.941^a^
Complex (≥3) chromosomal abnormality	46 (34.1%)	58 (37.2%)	0.668^a^

Monosomy = single chromosome loss; Trisomy = single chromosome gain; ^a^by Chi-square analysis.

**Figure 3:**
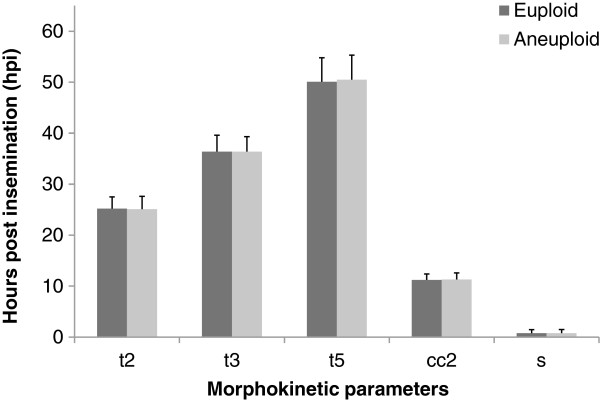
**Comparison of morphokinetic parameters of the early stages of embryonic development between euploid and aneuploid embryos in the time-lapse monitoring group.** t2 = time from insemination to 2 cells; t3 = time from insemination to 3 cells; t5 = time from insemination to 5 cells; cc2 = time between division to 2 cells and division to 3 cells; s2 = time between division to 3 cells and subsequent division to 4 cells; hpi = hours post insemination. Morphokinetic data were presented as mean ± SD. There were no significant differences between euploid and aneuploid embryos in each of the morphokinetic parameters (*p >*0.05 by Mann–Whitney test).

**Figure 4:**
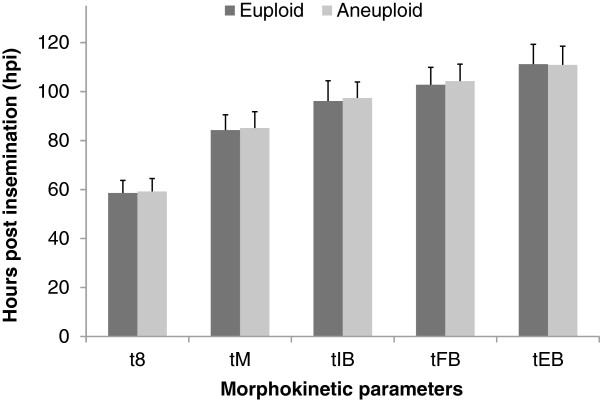
**Comparison of morphokinetic parameters of the later stages of embryonic development between euploid and aneuploid embryos in the time-lapse monitoring group.** t8 = time from insemination to 8 cells; tM = time from insemination to formation of a compact morula; tIB = time from insemination to initiation of blastulation; tFB = time from insemination to formation of a full blastocyst. tEB = time from insemination to formation of a expanded blastocyst. Morphokinetic data were presented as mean ± SD. There were no significant differences between euploid and aneuploid embryos in each of the morphokinetic parameters (*p >*0.05 by Mann–Whitney test).

The morphokinetic parameters of the early stages of embryonic development were compared between euploid and aneuploid embryos in the time-lapse monitoring group (Figure 3). There were no significant differences in the time from insemination to 5 cells (t5) between euploid and aneuploid embryos (50.1 ± 4.8 hpi vs. 50.5 ± 4.7 hpi, respectively, *p >*0.05). The time between division to 2 cells and division to 3 cells (cc2) of euploid embryos was similar to that of aneuploid embryos (11.2 ± 1.2 hpi vs. 11.3 ± 1.1 hpi, respectively, *p >*0.05). Moreover, the time between division to 3 cells and subsequent division to 4 cells (s2) was comparable between euploid and aneuploid embryos (0.77 ± 0.69 hpi vs. 0.78 ± 0.71 hpi, respectively, *p >*0.05). The morphokinetic parameters of the later stages of embryonic development were also compared between euploid and aneuploid embryos in the time-lapse monitoring group (Figure 4). The time from insemination to initiation of blastulation (tIB) was slightly delayed in aneuploid embryos compared to euploid embryos (97.4 ± 6.5 vs. 96.1 ± 6.8 hpi, respectively, *p* >0.05). The time from insemination to formation of a full blastocyst (tFB) of aneuploid embryos was also marginally slower than that of euploid embryos (104.3 ± 6.9 vs. 102.8 ± 7.2 hpi, respectively, *p* >0.05). Additionally, the time from insemination to formation of an expanded blastocyst (tEB) was comparable between aneuploid and euploid embryos (110.9 ± 8.1 vs. 111.2 ± 7.6 hpi, respectively, *p* >0.05). Importantly, none of the differences in morphokinetic parameters between euploid and aneuploid embryos approached statistical significance.

One to two euploid blastocysts within the most predictive morphokinetic parameters (Group A) or with the best morphological grade available (Group B) were selected for transfer to individual patients. As shown in Table 4, a total of 127 (92.0%) of the patients had euploid blastocysts for transfer while 8 (5.8%) of the patients ended with no euploid embryos available for transfer. In addition, 3 (2.2%) of the patients had embryos screened by array CGH first and then had the euploid blastocysts cryopreserved in order to avoid ovarian hyperstimulation syndrome (OHSS). Among the patients with euploid blastocysts for transfer in the time-lapse system group, 19 patients had single euploid blastocysts and 26 patients had double euploid blastocysts for transfer. In the conventional incubator group, 15 patients had single euploid blastocyst and 22 had double euploid blastocysts for transfer. The remaining 45 patients chose to have mixed embryos (one from the time-lapse system and one from the conventional incubator) transferred because they had one euploid blastocyst from each group available for transfer. These patients had prior history of unexplained recurrent pregnancy loss (n ≥ 2) and/or repeated implantation failure (n ≥ 3), and preferred to transfer two euploid blastocysts from both groups in order to increase the chances of a successful pregnancy. There were significant differences in clinical pregnancy rates between the time-lapse system (Group A) and the conventional incubator (Group B) (71.1% vs. 45.9%, respectively, *p* = 0.037). The observed implantation rate was significantly higher in Group A compared to Group B (66.2% vs. 42.4%, respectively, *p =* 0.011). A significant difference in ongoing pregnancy rate was also observed between Group A and Group B (68.9% vs. 40.5%, respectively, *p =* 0.019). However, there was no significant difference in miscarriage rate between Group A and Group B (3.1% vs. 11.8%, respectively, *p* = 0.273).

**Table 4:** Comparison of pregnancy and implantation outcomes between time-lapse (Group A) and conventional incubator (Group B), as well as the mixed embryo transfer

**Parameters**	**Group A**	**Group B**	**Mixed**	***p *value**
Patient with SET	19	15	n/a	
Patient with DET	26	22	45	
Clinical pregnancies after SET	10	5	n/a	
Clinical pregnancies after DET	21	11	24	
Clinical pregnancy rate	71.1%	45.9%	53.3%	0.037^a^
Implantation rate	66.2%	42.4%	47.8%	0.011^a^
Ongoing pregnancy rate	68.9%	40.5%	48.9%	0.019^a^
Pregnancy loss rate	3.1%	11.8%	8.3%	0.273^b^

SET = single embryo transfer; DET = double embryo transfer; Mixed = mixed embryo transfer (one from the time-lapse system and one from the conventional incubator); ^a^Group A vs. Group B, by Chi-square analysis; ^b^Group A vs. Group B, by Fisher’s exact test.

As previously described, blastocysts were evaluated and graded from 1 to 6 based on the morphological criteria before selecting for transfer in both groups. The morphological grades of transferred euploid blastocysts were compared between the time–lapse system (Group A) and the conventional incubator (Group B) (Table 5). There was no significant difference in the percentage of each morphological grade of the transferred euploid blastocysts between the two groups (*p >*0.05). Data in Table 6 further compare pregnancy and implantation outcomes between the euploid blastocysts with early initiation of blastulation (tIB < 96.1 hpi) and the euploid blastocysts with delayed initiation of blastulation (tIB ≥ 96.1 hpi) in the time-lapse system group. A non-significant increase in clinical pregnancy rate was observed in the euploid blastocysts with early initiation of blastulation compared to the euploid blastocysts with delayed initiation of blastulation (77.8% vs. 61.1%, respectively, *p >*0.05). Moreover, there was also an insignificant trend in which implantation rates increased in the euploid blastocysts with early initiation of blastulation compared to the euploid blastocysts with delayed initiation of blastulation (71.4% vs. 58.6%, respectively, *p >*0.05).

**Table 5:** Comparison of transferred euploid blastocysts at each grade between time-lapse system (Group A) and conventional incubator (Group B)

**Parameters**	**Group A**	**Group B**	***p *value**
Total transferred blastocysts	71	59	
Transferred blastocysts at Grade 3	4 (5.6%)	5 (8.5%)	0.731^a^
Transferred blastocysts at Grade 4	26 (36.6%)	27 (45.7%)	0.381^b^
Transferred blastocysts at Grade 5	38 (53.5%)	26 (44.1%)	0.369^b^
Transferred blastocysts at Grade 6	3 (4.2%)	1 (1.7%)	0.625^a^

^a^by Fisher’s exact test, ^b^by Chi-square analysis.

**Table 6:** Comparison of clinical pregnancy and implantation rates between euploid blastocysts with tIB < 96.1 hpi and euploid blastocysts with tIB ≥ 96.1 hpi in Group A

**Parameters**	**tIB < 96.1 hpi**	**tIB ≥ 96.1 hpi**	***p *value**
Patient with SET	12	7	
Patient with DET	15	11	
Clinical pregnancies after SET	8	3	
Clinical pregnancies after DET	13	8	
Clinical pregnancy rate	77.8%	61.1%	0.383^a^
Implantation rate	71.4%	58.6%	0.386^a^

tIB = time from insemination to initiation of blastulation; hpi = hours post insemination; SET = single embryo transfer; DET = double embryo transfer; ^a^Group A vs. Group B, by Chi-square analysis.

As shown in Table 7, there were a total of five miscarriages in five patients who had been clinically pregnant with gestational sac(s) and fetal heart beat(s) after embryo transfer: two in the conventional incubator (CI) group, one in the time-lapse system (TL) group and two in the mixed transfer (MIX) group. The average age of these patients was 38.2 years old (ranging from 37 to 39 years) with the clinical indications of unknown recurrent pregnancy loss, repeated implantation failure or previous aneuploid conceptions. Four of the patients (No. 2 to No. 5) had their products of conception analyzed while patient No. 1 had no cytogenetic analysis available due to an early spontaneous abortion. Among patients with cytogenetic analysis results, three patients (No. 2 to 4) had a singleton pregnancy loss and the cytogenetic analysis of the products of conception revealed normal karyotypes for all three patients. The leftover DNA samples from the blastocyst biopsy were reanalyzed by array CGH which revealed the same results as the cytogenetic analysis and the initial array CGH diagnosis (euploid), indicating that there could be a cause for the miscarriages other than aneuploidy in these patients. One of the patients (No. 5) had twin pregnancy loss, and the follow-up cytogenetic analysis of the products of conception revealed a mosaic 45X0/46XX and a trisomy 16 miscarriage. Reanalysis of the leftover DNA samples from the trophectoderm biopsy with array CGH revealed the same results as the initial array CGH diagnosis (euploid), suggesting mosaicism as the cause for the different results between the cytogenetic analysis (of the products of conception) and the array CGH testing (of the trophectoderm cells).

**Table 7:** List of patients with pregnancy loss in time-lapse system (TL), conventional incubator (CI) and mixed transfer (MIX) groups

**Patient’s ID**	**Age (years)**	**Clinical indication**	**Culture group**	**Pregnancy loss**	**Cytogenetic analysis**	**Array CGH result**
1	39	URPL	CI	Singleton	Unknown	Euploid
2	38	RIF	CI	Singleton	46XY	Euploid
3	39	URPL	TL	Singleton	46XX	Euploid
4	38	URPL	MIX	Singleton	46XY	Euploid
5	37	PAC	MIX	Twin	47XX + 16, 45X0/46XX	Euploid

URPL = unexplained recurrent pregnancy loss; RIF = repeated implantation failure; PAC = previous aneuploid conception; CI = conventional incubator; TL = time-lapse system; MIX = mixed embryo transfer (one embryo from the time-lapse system and another one from the conventional incubator).

## Discussion

The ultimate goal of preimplantation genetic screening and assisted reproductive treatment is to select one to two of the most competent embryos with normal chromosome compositions for transfer in order to maximize the chances of a successful pregnancy with delivery of a healthy baby while minimizing the incidence of miscarriages in each treatment cycle. Aneuploidy rates are extremely high in IVF patients, especially in those with unexplained recurrent pregnancy loss [44], repeated implantation failure [45] and/or previous aneuploid conceptions [46]. Recent studies with array CGH screening have demonstrated a significant improvement in pregnancy outcomes for PGS patients [37,64-66]. Meanwhile, recent advances in time-lapse culture and monitoring have provided new morphokinetic markers for selecting competent embryos for transfer [26,34]. In the current study, we have combined these two advanced technologies available in our IVF clinics to provide the advantage of selecting competent blastocysts for transfer and thereby maximizing the chances of a successful pregnancy for our PGS patients. There were significant differences in clinical pregnancy rates between the time-lapse system (Group A) and the conventional incubator (Group B) (71.1% vs. 45.9%, respectively, *p =* 0.037). Moreover, the implantation rate was higher in Group A compared to Group B (66.2% vs. 42.4%, respectively, *p =* 0.011). A significant difference in the ongoing pregnancy rate was also observed between Group A and Group B (68.9% vs. 40.5%, respectively, *p =* 0.019). Collectively, our data show the distinct benefits of combining time-lapse monitoring and array CGH testing to select competent blastocysts for transfer for patients undergoing preimplantation screening. A recent retrospective analysis of a large number of IVF treatment cycles (n = 7305) also concluded that monitoring and selecting embryos in the time-lapse system significantly improved clinical pregnancy and implantation rates compared to the conventional incubator [34].

Compared to previous reports, our current study has multiple advantages with regard to studying the clinical benefits of combining time-lapse monitoring and array CGH testing to select competent blastocysts for transfer in PGS patients. First, ploidy was determined with array CGH testing, and selection of embryos for transfer was primarily based on the array CGH results in both time-lapse system and conventional incubator groups in order to ensure that only euploid embryos were selected for transfer to patients. In the time-lapse system group, the morphokinetic markers within the most predictive parameters were the secondary criterion for selection when multiple euploid blastocysts were recognized from individual patients. In the conventional incubator group, morphological grading by microscopic evaluation was the secondary criterion for selection when multiple euploid blastocysts were available. However, in previous studies comparing the time-lapse system and the conventional incubator, ploidy of the transferred embryos had not been determined before the embryos were selected for transfer. Lack of chromosomal screening may lead to transfer of euploid and/or aneuploid embryos to patients, producing inconsistent data and conflicting pregnancy outcomes [32-34,36]. Second, in our prospective study, a sibling oocyte model was designated so that the patients served as their own control, and much larger numbers of MII oocytes (n = 1163) were included in order to draw a firmer statistical conclusion compared to the earlier time-lapse studies with sibling oocytes [32,33]. Moreover, a relatively larger number (n = 138) of younger patients (at a mean age of 36.6 ± 2.4 year ranging 28 to 39 years) was included in the present study in order to avoid the effects of advanced maternal age on morphokinetic parameters and chromosomal status of embryos when compared to previous research exploring the relationship between morphokinetic parameters and aneuploidy [20]. It has been well documented that the aneuploidy rate increases with maternal age [37-41,44-46], especially at advanced maternal ages [11,70]. Recent studies have also revealed that maternal age is one of the major confounding factors affecting clinical outcomes as related to morphokinetic parameters of human embryos that were cultured and monitored in time-lapse systems [34,36]. Furthermore, in the present study, the time-lapse system was closely monitored and constantly operated with reduced oxygen tension (5%). In the previous studies comparing embryo culture in the time-lapse system and the conventional incubator, however, embryos were entirely cultured under atmospheric oxygen concentration (20%) and the pregnancy and implantation outcomes were not optimized in the time-lapse system group [32,33]. The significance of culturing oocytes and embryos under low oxygen tension has been well documented in mammalian species including humans [16,18,81-83]. Studies with various species of mammals have demonstrated that the concentration of oxygen inside the uterus and oviduct usually falls in the ranges of 2-8%. Improved clinical pregnancy, implantation and live birth rates have also been reported after the use of reduced oxygen tension for embryonic culture to the blastocyst stage [16,82,84]. These results are associated with a reduction of the harmful effects of reactive oxygen species (ROS). The increase in the generation and accumulation of ROS is associated with various types of cell damage including DNA fragmentation, altered gene expression, and organelle and membrane disturbances in oocytes and embryos [81,82]. Consequently, interrupted or delayed embryonic development, apoptosis or health impairment during pregnancy can be observed in embryos cultured under atmospheric conditions [83,84]. In the current study, clinical pregnancy, implantation and ongoing pregnancy rates were significantly improved in the time-lapse system with reduced oxygen concentration compared to the conventional incubator with atmospheric oxygen concentration. Collectively, our data suggest that the use of time-lapse culture and monitoring with low oxygen tension may improve clinical and implantation outcomes for PGS patients. Finally, the temperature was strictly monitored and controlled in the time-lapse system during the entire period of the current study. In addition, all fertilized oocytes were cultured to the blastocyst stage in the continuous single culture medium (CSC, Irvine Scientific, Irvine, USA) to avoid sudden changes in culture conditions, especially temperature fluctuation. Adverse effects of temperature fluctuation on the meiotic spindle have been well documented in various mammalian species [18]. Transient cooling to room temperature can cause irreversible disruption of the meiotic spindle in human oocytes and embryos [19]. Such disruption may, in turn, result in the elevated levels of aneuploidy in human oocyte and embryos, especially when embryos are handled outside of the incubator during medium change and evaluation, where the earlier PGS studies were performed [53-57].

By combining these two advanced technologies, this prospective study extends prior research where either time-lapse monitoring or array CGH screening alone was used for evaluation and selection of competent embryos for transfer. To the best of our knowledge, this is the first prospective study with sibling oocytes to apply both time-lapse monitoring and array CGH testing to select competent blastocysts for transfer in patients undergoing preimplantation genetic screening. Our research contributes new array CGH and time-lapse evaluation data, assuring the importance of selecting competent embryos for transfer in the PGS patients with various clinical indications. The extent of aneuploidy in human embryos can be extensive [37-46], although this rate is typically lower in embryos at blastocyst stage [43,60]. This prospective study provides further evidence of substantial chromosomal abnormalities in apparently normal blastocysts inside or outside of range of the most predictive morphokinetic parameters, including monosomy, trisomy, dual and complex aneuploidy [11,12,20,60]. Our data also confirmed the previous observation that morphological evaluation should not be solely relied upon in the selection of competent embryos for transfer [11,12]. Moreover, there were no significant differences in any of the morphokinetic parameters of the early embryonic development between euploid and aneuploid embryos, although there was a slight delay in some of the morphokinetic parameters at the late stage of embryonic development in aneuploid embryos compared to euploid embryos. Additionally, there was a non-significant trend in which clinical pregnancy and implantation rates increased in the euploid blastocysts with early initiation of blastulation compared to the euploid blastocysts with delayed initiation of blastulation. These data suggest that ploidy of the transferred blastocysts may be likely the primary factor for determining the clinical pregnancy and implantation outcomes in patients undergoing preimplantation genetic screening, while morphokinetic markers of the last stages of embryonic development (e.g. tIB) may be used as a complementary system [20] to array CGH for embryonic selection. Thus, the combination of time-lapse monitoring and array CGH testing should be recommended for PGS patients to maximize the chances of successful pregnancies and to minimize the incidences of harmful miscarriages.

Several limitations in our prospective study should be addressed. First, although the combination of time-lapse evaluation and array CGH screening displays distinct benefits for many patients undergoing preimplantation genetic screening, this approach is not for all IVF patients with various clinical indications, especially those with diminished ovarian reserve or poor stimulation responders. The improved implantation and ongoing pregnancy rates in the time-lapse monitoring group noted here may not necessarily apply to patients in all age groups, especially those over 40 years old. Moreover, the observed difference in results between array CGH testing of the trophectoderm cells and the cytogenetic analysis of the products of conception suggests that mosaicism may be the cause of the misdiagnosis of a small proportion of human embryos at the blastocyst stages [37,85], although this mosaicism rate is generally believed to be lower than that of embryos at cleavage stages [37,43,60,85]. Additionally, there was a non-significant trend in which the rate of pregnancy loss decreased in the time-lapse system compared to the conventional incubator (3.1% vs. 11.8%, respectively, *p* = 0.273). This observation may be due to the cumulative sample size being insufficient to detect a significant difference in this category. Finally, potential epigenetic effects as related to external factors such as stimulation protocol, culture media, light exposure, incubation conditions and manipulation of embryos remain relatively unknown [86,87]. Further prospective clinical trials with a larger scale of randomized samples may be helpful in clarifying these issues.

## Conclusion

In conclusion, our results represent the first prospective investigation using sibling oocytes to evaluate the efficiency of selecting competent blastocysts for transfer by combining time-lapse monitoring and array CGH testing for patients undergoing preimplantation genetic screening. Our data clearly demonstrate that the combination of these two advanced technologies to select competent blastocysts for transfer results in improved implantation and ongoing pregnancy rates for PGS patients. Further randomized clinical trials with a larger sample size are planned to verify these initial findings and to confirm the clinical benefits of combining time-lapse monitoring and array CGH testing for PGS patients.

## Competing interests

The authors declare that they have no competing interests.

## Authors’ contributions

ZY and JL conceived and designed the study. ZY is the Scientific Director in charge of ART and PGD programs. JZ, JL, SS, KY and RS are fertility specialists in charge of the clinical programs. ZY wrote and edited the manuscript. XL and KY are responsible for data mining and statistical analysis. All authors read and approved the final manuscript.

## Pre-publication history

The pre-publication history for this paper can be accessed here:

http://www.biomedcentral.com/1755-8794/7/38/prepub

## References

[B1] Edwards RG Purdy JM Steptoe PC Walters DE The growth of human preimplantation embryos in vitro Am J Obstet Gynecol 1981 7 408 416 7282823 10.1016/0002-9378(81)90603-7

[B2] Racowsky C Vernon M Mayer J Ball GD Behr B Pomeroy KO Wininger D Gibbons W Conaghan J Stern JE Standardization of grading embryo morphology J Assist Reprod Genet 2010 7 437 439 20532975 10.1007/s10815-010-9443-2 PMC2941588

[B3] Alpha Scientists in Reproductive Medicine and ESHRE Special Interest Group of Embryology The Istanbul consensus workshop on embryo assessment: proceedings of an expert meeting Hum Reprod 2011 7 1270 1283 10.1093/humrep/der037 21502182

[B4] Cummins JM Breen TM Harrison KL Shaw JM Wilson LM Hennessey JF A formula for scoring human embryo growth rates in in vitro fertilization: its value in predicting pregnancy and in comparison with visual estimates of embryo quality J In Vitro Fert Embryo Transf 1986 7 284 295 10.1007/BF01133388 3783014

[B5] Giorgetti C Terriou P Auquier P Hans E Spach JL Salzmann J Roulier R Embryo score to predict implantation after in-vitro fertilization: based on 957 single embryo transfers Hum Reprod 1995 7 2427 2431 8530679 10.1093/oxfordjournals.humrep.a136312

[B6] Racowsky C Ohno-Machado L Kim J Biggers JD Is there an advantage in scoring early embryos on more than one day? Hum Reprod 2009 7 2104 2113 19493872 10.1093/humrep/dep198 PMC2727402

[B7] Racowsky C Combelles CMH Nureddin A Pan Y Finn A Miles L Gale S O'Leary T Jackson KV Day 3 and day 5 morphological predictors of embryo viability Reprod Biomed Online 2003 7 323 331 12735868 10.1016/s1472-6483(10)61852-4

[B8] Scott L Alvero R Leondires M Miller B The morphology of human pronuclear embryos is positively related to blastocyst development and implantation Hum Reprod 2000 7 2394 2403 11056141 10.1093/humrep/15.11.2394

[B9] Chen C Kattera S Comparison of pronuclear zygote morphology and early cleavage status of zygotes as additional criteria in the selection of day 3 embryos: a randomized study Fertil Steril 2006 7 347 352 16595210 10.1016/j.fertnstert.2005.07.1319

[B10] Gardner DK Schoolcraft WB Culture and transfer of human blastocysts Curr Opin Obstet Gynecol 1999 7 307 311 10369209 10.1097/00001703-199906000-00013

[B11] Alfarawati S Fragouli E Colls P Stevens J Gutierrez-Mateo C Schoolcraft WB Katz-Jaffe MG Wells D The relationship between blastocyst morphology, chromosomal abnormality, and embryo gender Fertil Steril 2011 7 520 524 20537630 10.1016/j.fertnstert.2010.04.003

[B12] Yang Z Liu J Collins GS Salem SA Liu X Lyle SS Peck AC Sills ES Salem RD Selection of single blastocysts for fresh transfer via standard morphology assessment alone and with array CGH for good prognosis IVF patients: results from a randomized pilot study Mol Cytogenet 2012 7 24 22551456 10.1186/1755-8166-5-24 PMC3403960

[B13] Liu J Sills ES Yang Z Salem SA Rahil T Collins GS Liu X Salem RD Array comparative genomic hybridization screening in IVF significantly reduces number of embryos available for cryopreservation Clin Exp Reprod Med 2012 7 52 57 22816070 10.5653/cerm.2012.39.2.52 PMC3398117

[B14] Yang Z Salem SA Liu X Kuang Y Salem RD Liu J Selection of euploid blastocysts for cryopreservation with array comparative genomic hybridization (aCGH) results in increased implantation rates in subsequent frozen and thawed embryo transfer cycles Mol Cytogenet 2013 7 32 23937723 10.1186/1755-8166-6-32 PMC3766007

[B15] Zhang JQ Li XL Peng Y Guo X Heng BC Tong GQ Reduction in exposure of human embryos outside the incubator enhances embryo quality and blastulation rate Reprod Biomed Online 2010 7 510 515 20129824 10.1016/j.rbmo.2009.12.027

[B16] Calzi F Papaleo E Rabellotti E Ottolina J Vailati S Vigano P Candiani M Exposure of embryos to oxygen at low concentration in a cleavage stage transfer program: reproductive outcomes in a time-series analysis Clin Lab 2012 7 997 1003 23163116

[B17] Swain JE Optimizing the culture environment in the IVF laboratory: impact of pH and buffer capacity on gamete and embryo quality Reprod Biomed Online 2010 7 6 16 20570214 10.1016/j.rbmo.2010.03.012

[B18] Gomes Sobrinho DB Oliveira JBA Petersen CG Mauri AL Silva LFI Massaro FC Baruffi RLR Cavagna M Franco JG IVF/ICSI outcomes after culture of human embryos at low oxygen tension: a meta-analysis Reprod Biol Endocrinol 2011 7 143 22044493 10.1186/1477-7827-9-143 PMC3229451

[B19] Pickering SJ Braude PR Johnson MH Cant A Currie J Transient cooling to room temperature can cause irreversible disruption of the meiotic spindle in the human oocyte Fertil Steril 1990 7 102 108 2358076 10.1016/s0015-0282(16)53644-9

[B20] Campbell A Fishel S Bowman N Duffy S Sedler M Hickman CFL Modelling a risk classification of aneuploidy in human embryos using non-invasive morphokinetics Reprod Biomed Online 2013 7 477 485 23518033 10.1016/j.rbmo.2013.02.006

[B21] Campbell A Fishel S Bowman N Duffy S Sedler M Thornton S Retrospective analysis of outcomes after IVF using an aneuploidy risk model derived from time-lapse imaging without PGS Reprod Biomed Online 2013 7 140 146 23683847 10.1016/j.rbmo.2013.04.013

[B22] Payne D Flaherty SP Barry MF Matthews CD Preliminary observations on polar body extrusion and pronuclear formation in human oocytes using time-lapse video cinematography Hum Reprod 1997 7 532 541 9130755 10.1093/humrep/12.3.532

[B23] Mio Y Maeda K Time-lapse cinematography of dynamic changes occurring during in vitro development of human embryos Am J Obstet Gynecol 2008 7 1 5 10.1016/j.ajog.2008.07.023 18823872

[B24] Lemmen JG Agerholm I Ziebe S Kinetic markers of human embryo quality using time-lapse recordings of IVF/ICSI-fertilized oocytes Reprod Biomed Online 2008 7 385 391 18765009 10.1016/s1472-6483(10)60222-2

[B25] Arav A Aroyo A Yavin S Roth Z Prediction of embryonic developmental competence by time-lapse observation and 'shortest-half' analysis Reprod Biomed Online 2008 7 669 675 18983751 10.1016/s1472-6483(10)60314-8

[B26] Meseguer M Herrero J Tejera A Hilligsoe KM Ramsing NB Remohi J The use of morphokinetics as a predictor of embryo implantation Hum Reprod 2011 7 2658 2671 21828117 10.1093/humrep/der256

[B27] Rubio I Kuhlmann R Agerholm I Kirk J Herrero J Escriba M-J Bellver J Meseguer M Limited implantation success of direct-cleaved human zygotes: a time-lapse study Fertil Steril 2012 7 1458 1463 22925687 10.1016/j.fertnstert.2012.07.1135

[B28] Wong CC Loewke KE Bossert NL Behr B De Jonge CJ Baer TM Reijo Pera RA Non-invasive imaging of human embryos before embryonic genome activation predicts development to the blastocyst stage Nat Biotechnol 2010 7 1115 1121 20890283 10.1038/nbt.1686

[B29] Pribenszky C Matyas S Kovacs P Losonczi E Zadori J Vajta G Pregnancy achieved by transfer of a single blastocyst selected by time-lapse monitoring Reprod Biomed Online 2010 7 533 536 20638906 10.1016/j.rbmo.2010.04.015

[B30] Ciray HN Aksoy T Goktas C Ozturk B Bahceci M Time-lapse evaluation of human embryo development in single versus sequential culture media–a sibling oocyte study J Assist Reprod Genet 2012 7 891 900 22714134 10.1007/s10815-012-9818-7 PMC3463674

[B31] Kirkegaard K Hindkjaer JJ Ingerslev HJ Human embryonic development after blastomere removal: a time-lapse analysis Hum Reprod 2012 7 97 105 22081251 10.1093/humrep/der382

[B32] Cruz M Gadea B Garrido N Pedersen KS Martinez M Perez-Cano I Munoz M Meseguer M Embryo quality, blastocyst and ongoing pregnancy rates in oocyte donation patients whose embryos were monitored by time-lapse imaging J Assist Reprod Genet 2011 7 569 573 21394522 10.1007/s10815-011-9549-1 PMC3162049

[B33] Kirkegaard K Hindkjaer JJ Grondahl ML Kesmodel US Ingerslev HJ A randomized clinical trial comparing embryo culture in a conventional incubator with a time-lapse incubator J Assist Reprod Genet 2012 7 565 572 22460082 10.1007/s10815-012-9750-x PMC3370049

[B34] Meseguer M Rubio I Cruz M Basile N Marcos J Requena A Embryo incubation and selection in a time-lapse monitoring system improves pregnancy outcome compared with a standard incubator: a retrospective cohort study Fertil Steril 2012 7 1481 1489 22975113 10.1016/j.fertnstert.2012.08.016

[B35] Conaghan J Chen AA Willman SP Ivani K Chenette PE Boostanfar R Baker VL Adamson G Abusief M Gvakharia M Loewke KE Shen S Improving embryo selection using a computer-automated time-lapse image analysis test plus day 3 morphology: results from a prospective multicenter trial Fertil Steril 2013 7 412 419 23721712 10.1016/j.fertnstert.2013.04.021

[B36] Kirkegaard K Kesmodel US Hindkjær JJ Ingerslev HJ Time-lapse as predictors of blastocyst development and pregnancy outcome in embryos from good prognosis patients: a prospective cohory study Hum Reprod 2013 7 2643 2651 23900207 10.1093/humrep/det300

[B37] Hodes-Wertz B Grifo J Ghadir S Kaplan B Laskin CA Glassner M Munne S Idiopathic recurrent miscarriage is caused mostly by aneuploid embryos Fertil Steril 2012 7 675 680 22683012 10.1016/j.fertnstert.2012.05.025

[B38] Wilton L Preimplantation genetic diagnosis and chromosome analysis of blastomeres using comparative genomic hybridization Hum Reprod Update 2005 7 33 41 15569702 10.1093/humupd/dmh050

[B39] Munne S Alikani M Tomkin G Grifo J Cohen J Embryo morphology, developmental rates, and maternal age are correlated with chromosome abnormalities Fertil Steril 1995 7 382 391 7615118

[B40] Hassold T Hunt P Maternal age and chromosomally abnormal pregnancies: what we know and what we wish we knew Curr Opin Pediatr 2009 7 703 708 19881348 10.1097/MOP.0b013e328332c6ab PMC2894811

[B41] Kuliev A Cieslak J Verlinsky Y Frequency and distribution of chromosome abnormalities in human oocytes Cytogenet Genome Res 2005 7 193 198 16192694 10.1159/000086889

[B42] Mantzouratou A Delhanty JDA Aneuploidy in the human cleavage stage embryo Cytogenet Genome Res 2011 7 141 148 21293113 10.1159/000323794

[B43] Fragouli E Wells D Aneuploidy in the human blastocyst Cytogenet Genome Res 2011 7 149 159 21252488 10.1159/000323500

[B44] Rubio C Simon C Vidal F Rodrigo L Pehlivan T Remohi J Pellicer A Chromosomal abnormalities and embryo development in recurrent miscarriage couples Hum Reprod 2003 7 182 188 12525464 10.1093/humrep/deg015

[B45] Voullaire L Wilton L McBain J Callaghan T Williamson R Chromosome abnormalities identified by comparative genomic hybridization in embryos from women with repeated implantation failure Mol Hum Reprod 2002 7 1035 1041 12397217 10.1093/molehr/8.11.1035

[B46] Munne S Sandalinas M Magli C Gianaroli L Cohen J Warburton D Increased rate of aneuploid embryos in young women with previous aneuploid conceptions Prenat Diagn 2004 7 638 643 15305354 10.1002/pd.957

[B47] Handyside AH Kontogianni EH Hardy K Winston RM Pregnancies from biopsied human preimplantation embryos sexed by Y-specific DNA amplification Nature 1990 7 768 770 2330030 10.1038/344768a0

[B48] Handyside AH Lesko JG Tarin JJ Winston RM Hughes MR Birth of a normal girl after in vitro fertilization and preimplantation diagnostic testing for cystic fibrosis N Engl J Med 1992 7 905 909 1381054 10.1056/NEJM199209243271301

[B49] Liu J Lissens W Devroey P Liebaers I Van Steirteghem AC Efficiency of polymerase chain reaction assay for cystic fibrosis in single human blastomeres according to the presence or absence of nuclei Fertil Steril 1993 7 815 819 8458502 10.1016/s0015-0282(16)55865-8

[B50] Delhanty JD Griffin DK Handyside AH Harper J Atkinson GH Pieters MH Winston RM Detection of aneuploidy and chromosomal mosaicism in human embryos during preimplantation sex determination by fluorescent in situ hybridisation, (FISH) Hum Mol Genet 1993 7 1183 1185 8401499 10.1093/hmg/2.8.1183

[B51] Munne S Lee A Rosenwaks Z Grifo J Cohen J Diagnosis of major chromosome aneuploidies in human preimplantation embryos Hum Reprod 1993 7 2185 2191 8150922 10.1093/oxfordjournals.humrep.a138001

[B52] Harper JC Delhanty JD Detection of chromosomal abnormalities in human preimplantation embryos using FISH J Assist Reprod Genet 1996 7 137 139 8688586 10.1007/BF02072535

[B53] Gianaroli L Magli MC Ferraretti AP Fiorentino A Garrisi J Munne S Preimplantation genetic diagnosis increases the implantation rate in human in vitro fertilization by avoiding the transfer of chromosomally abnormal embryos Fertil Steril 1997 7 1128 1131 9418710 10.1016/s0015-0282(97)00412-3

[B54] Staessen C Verpoest W Donoso P Haentjens P Van der Elst J Liebaers I Devroey P Preimplantation genetic screening does not improve delivery rate in women under the age of 36 following single-embryo transfer Hum Reprod 2008 7 2818 2825 18930977 10.1093/humrep/den367

[B55] Hardarson T Hanson C Lundin K Hillensjo T Nilsson L Stevic J Reismer E Borg K Wikland M Bergh C Preimplantation genetic screening in women of advanced maternal age caused a decrease in clinical pregnancy rate: a randomized controlled trial Hum Reprod 2008 7 2806 2812 18583331 10.1093/humrep/den217

[B56] Schoolcraft WB Katz-Jaffe MG Stevens J Rawlins M Munne S Preimplantation aneuploidy testing for infertile patients of advanced maternal age: a randomized prospective trial Fertil Steril 2009 7 157 162 18692827 10.1016/j.fertnstert.2008.05.029

[B57] Debrock S Melotte C Spiessens C Peeraer K Vanneste E Meeuwis L Meuleman C Frijns J-P Vermeesch JR D'Hooghe TM Preimplantation genetic screening for aneuploidy of embryos after in vitro fertilization in women aged at least 35 years: a prospective randomized trial Fertil Steril 2010 7 364 373 19249029 10.1016/j.fertnstert.2008.10.072

[B58] Wells D Delhanty JD Comprehensive chromosomal analysis of human preimplantation embryos using whole genome amplification and single cell comparative genomic hybridization Mol Hum Reprod 2000 7 1055 1062 11044470 10.1093/molehr/6.11.1055

[B59] Voullaire L Wilton L Slater H Williamson R Detection of aneuploidy in single cells using comparative genomic hybridization Prenat Diagn 1999 7 846 851 10521843

[B60] Fragouli E Lenzi M Ross R Katz-Jaffe M Schoolcraft WB Wells D Comprehensive molecular cytogenetic analysis of the human blastocyst stage Hum Reprod 2008 7 2596 2608 18664475 10.1093/humrep/den287

[B61] Sher G Keskintepe L Keskintepe M Maassarani G Tortoriello D Brody S Genetic analysis of human embryos by metaphase comparative genomic hybridization (mCGH) improves efficiency of IVF by increasing embryo implantation rate and reducing multiple pregnancies and spontaneous miscarriages Fertil Steril 2009 7 1886 1894 19135663 10.1016/j.fertnstert.2008.11.029

[B62] Schoolcraft WB Fragouli E Stevens J Munne S Katz-Jaffe MG Wells D Clinical application of comprehensive chromosomal screening at the blastocyst stage Fertil Steril 2010 7 1700 1706 19939370 10.1016/j.fertnstert.2009.10.015

[B63] Gutierrez-Mateo C Colls P Sanchez-Garcia J Escudero T Prates R Ketterson K Wells D Munne S Validation of microarray comparative genomic hybridization for comprehensive chromosome analysis of embryos Fertil Steril 2011 7 953 958 20971462 10.1016/j.fertnstert.2010.09.010

[B64] Fishel S Gordon A Lynch C Dowell K Ndukwe G Kelada E Thornton S Jenner L Cater E Brown A Garcia-Benardo J Live birth after polar body array comparative genomic hybridization prediction of embryo ploidy-the future of IVF? Fertil Steril 2010 7 1006 e7-e10 19939361 10.1016/j.fertnstert.2009.09.055

[B65] Alfarawati S Fragouli E Colls P Wells D First births after preimplantation genetic diagnosis of structural chromosome abnormalities using comparative genomic hybridization and microarray analysis Hum Reprod 2011 7 1560 1574 21447693 10.1093/humrep/der068

[B66] Fiorentino F Spizzichino L Bono S Biricik A Kokkali G Rienzi L Ubaldi FM Iammarrone E Gordon A Pantos K PGD for reciprocal and Robertsonian translocations using array comparative genomic hybridization Hum Reprod 2011 7 1925 1935 21489979 10.1093/humrep/der082

[B67] Gabriel AS Thornhill AR Ottolini CS Gordon A Brown APC Taylor J Bennett K Handyside A Griffin DK Array comparative genomic hybridisation on first polar bodies suggests that non-disjunction is not the predominant mechanism leading to aneuploidy in humans J Med Genet 2011 7 433 437 21617258 10.1136/jmg.2010.088070

[B68] Geraedts J Montag M Magli MC Repping S Handyside A Staessen C Harper J Schmutzler A Collins J Goossens V van der Ven H Vesela K Gianaroli L Polar body array CGH for prediction of the status of the corresponding oocyte. Part I: clinical results Hum Reprod 2011 7 3173 3180 21908463 10.1093/humrep/der294 PMC3196878

[B69] Fragouli E Alfarawati S Daphnis DD Goodall NN Mania A Griffiths T Gordon A Wells D Cytogenetic analysis of human blastocysts with the use of FISH, CGH and aCGH: scientific data and technical evaluation Hum Reprod 2011 7 480 490 21147821 10.1093/humrep/deq344

[B70] Liu J Wang W Sun X Liu L Jin H Li M Witz C Williams D Griffith J Skorupski J Haddad G Gill J DNA microarray reveals that high proportions of human blastocysts from women of advanced maternal age are aneuploid and mosaic Biol Reprod 2012 7 148 148 23136294 10.1095/biolreprod.112.103192

[B71] Capalbo A Bono S Spizzichino L Biricik A Baldi M Colamaria S Ubaldi FM Rienzi L Fiorentino F Sequential comprehensive chromosome analysis on polar bodies, blastomeres and trophoblast: insights into female meiotic errors and chromosomal segregation in the preimplantation window of embryo development Hum Reprod 2013 7 509 518 23148203 10.1093/humrep/des394

[B72] Handyside AH PGD and aneuploidy screening for 24 chromosomes by genome-wide SNP analysis: seeing the wood and the trees Reprod Biomed Online 2011 7 686 691 22033395 10.1016/j.rbmo.2011.09.012

[B73] Treff NR Su J Tao X Levy B Scott RT Accurate single cell 24 chromosome aneuploidy screening using whole genome amplification and single nucleotide polymorphism microarrays Fertil Steril 2010 7 2017 2021 20188357 10.1016/j.fertnstert.2010.01.052

[B74] Northrop LE Treff NR Levy B Scott RT Jr SNP microarray-based 24 chromosome aneuploidy screening demonstrates that cleavage-stage FISH poorly predicts aneuploidy in embryos that develop to morphologically normal blastocysts Mol Hum Reprod 2010 7 590 600 20479065 10.1093/molehr/gaq037 PMC2907218

[B75] Johnson DS Gemelos G Baner J Ryan A Cinnioglu C Banjevic M Ross R Alper M Barrett B Frederick J Potter D Behr B Rabinowitz M Preclinical validation of a microarray method for full molecular karyotyping of blastomeres in a 24-h protocol Hum Reprod 2010 7 1066 1075 20100701 10.1093/humrep/dep452 PMC2839907

[B76] Lathi RB Massie JAM Gilani M Milki AA Westphal LM Baker VL Behr B Outcomes of trophectoderm biopsy on cryopreserved blastocysts: a case series Reprod Biomed Online 2012 7 504 507 22985500 10.1016/j.rbmo.2012.06.021

[B77] Scott RT Jr Upham KM Forman EJ Hong KH Scott KL Talor D Tao X Treff NR Blastocyst biopsy with comprehensive chromosome screening and fresh embryo transfer significantly increases in vitro fertilization implantation and delivery rates: a randomized controlled trial Fertil Sterilin press 10.1016/j.fertnstert.2013.04.035 23731996

[B78] Scott RT Ferry K Su J Tao X Scott K Treff NR Comprehensive chromosome screening is highly predictive of the reproductive potential of human embryos: a prospective, blinded, nonselection study Fertil Steril 2012 7 870 875 22305103 10.1016/j.fertnstert.2012.01.104

[B79] Treff NR Tao X Ferry KM Su J Taylor D Scott RT Development and validation of an accurate quantitative real-time polymerase chain reaction-based assay for human blastocyst comprehensive chromosomal aneuploidy screening Fertil Steril 2012 7 819 824 22342859 10.1016/j.fertnstert.2012.01.115

[B80] Forman EJ Tao X Ferry KM Taylor D Treff NR Scott RT Single embryo transfer with comprehensive chromosome screening results in improved ongoing pregnancy rates and decreased miscarriage rates Hum Reprod 2012 7 1217 1222 22343551 10.1093/humrep/des020 PMC3303493

[B81] Catt JW Henman M Toxic effects of oxygen on human embryo development Hum Reprod 2000 7 Suppl 2 199 206 11041525 10.1093/humrep/15.suppl_2.199

[B82] Meintjes M Chantilis SJ Douglas JD Rodriguez AJ Guerami AR Bookout DM Barnett BD Madden JD A controlled randomized trial evaluating the effect of lowered incubator oxygen tension on live births in a predominantly blastocyst transfer program Hum Reprod 2009 7 300 307 18927130 10.1093/humrep/den368

[B83] Yang HW Hwang KJ Kwon HC Kim HS Choi KW Oh KS Detection of reactive oxygen species (ROS) and apoptosis in human fragmented embryos Hum Reprod 1998 7 998 1002 9619561 10.1093/humrep/13.4.998

[B84] Kovacic B Vlaisavljevic V Influence of atmospheric versus reduced oxygen concentration on development of human blastocysts in vitro: a prospective study on sibling oocytes Reprod Biomed Online 2008 7 229 236 18681997 10.1016/s1472-6483(10)60199-x

[B85] Capalbo A Wright G Elliot T Ubaldi FM Rienzi L Nagy ZP FISH reananlysis of inner cell mass and trophectoderm samples of previously array-CGH screened blastocysts shows high accuracy of diagnosis and no major diagnostic impact of mosaicism at the blastocyst stage Hum Reprod 2013 7 2298 2307 23739221 10.1093/humrep/det245

[B86] Niemitz EL Feinberg AP Epigenetics and assisted reproductive technology: a call for investigation Am J Hum Genet 2004 7 599 609 14991528 10.1086/382897 PMC1181938

[B87] Horsthemke B Ludwig M Assisted reproduction: the epigenetic perspective Hum Reprod Update 2005 7 473 482 15994847 10.1093/humupd/dmi022

